# The critical role of health policy and management in epidemic control: COVID-19 and beyond

**DOI:** 10.1016/j.imj.2024.100151

**Published:** 2024-11-09

**Authors:** Zeyu Zhang, You Wu

**Affiliations:** a Institute for Hospital Management, Tsinghua University, Beijing 100084, China; b School of Basic Medical Sciences, Tsinghua University, Beijing 100084, China

## 1 Introduction

The COVID-19 pandemic disrupted global health systems and economies and exposed not only the fragility of public health infrastructures but also the resilience of health management and governance systems. Declaration of the pandemic's end by the World Health Organization (WHO) does not mean that emerging infectious diseases such as COVID-19 no longer threaten human health. Experiences from dealing with COVID-19 are valuable to improve preparedness for future public health emergencies. Although much attention has been rightfully focused on the virological and clinical dimensions of the pandemic, the substantial contributions made by health policy and public health management are often overlooked. In this editorial, we propose a unique lens into the influence of policymaking on management strategies and public health practices that shaped epidemic responses during this unprecedented period, serving to strengthen public health systems and prepare for potential public health emergencies of international concern (PHEIC).

## 2 Evolving clinical management protocols

With rapidly evolving transmission patterns and mutations, the dynamic nature of COVID-19 required constant updating of clinical management. The WHO promptly issued revised recommendations and interim guidance on the clinical management of COVID-19 [1]. At country level, the evolution of COVID-19 diagnosis and treatment protocols in China provides a prime example of adaptability [2]. In issuing up to 10 versions of treatment guidelines, Chinese health authorities demonstrated the importance of agility in policymaking, ensuring that the latest scientific findings were incorporated into clinical practice as soon as they became available. These updates were not simply technical revisions but also reflected a deeper understanding of socioeconomic factors at play [3]. Studies from the perspective of health care management revealed the importance of monitoring the burden on health care systems and the corresponding planning regarding hospital capacities, nosocomial infections, and discharge criteria to minimize impacts [4,5].

## 3 Optimization of prevention and control measures

Pandemic management involves various phases, including preparedness, response, and recovery. Policies should be adjusted and optimized according to the evolution of a pandemic to balance control and socioeconomic development. Precision in public health responses based on the characteristics of each phase is vital.

During the early outbreak period, establishing the epidemic curve is valuable to provide information for authorities to make swift decisions [6]. During the early phase of the COVID-19 pandemic, limited knowledge regarding this novel virus and unprepared health care systems drove the implementation of restrictions on travel and in-person contact, which inevitably hindered socioeconomic activities. Globally, examples across countries worldwide illustrate the varied approaches used to integrate economic aid and resilience planning during that time (Table 1).

**Table 1:** COVID-19 control measures balanced with local economic recovery in selected cities^⁎^.

Region and city	COVID-19 control measures	Economic impact	Recovery and response efforts
Africa			
Accra, Ghana	Traffic restrictions, phased re-openings, health-related restrictions	IGF decreased by 35 %, high business closures	Economic aid partnerships, soft loans for businesses, food distribution, resilience strategy incorporated into city development plan
Arua, Uganda	Traffic restrictions, curfews, public health protocols	Reduced business income, job loss, transport costs high	Rent waivers, agricultural sector recovery, business development support, and service digitalization
Harare, Zimbabwe	Traffic restrictions with intermittent extensions, sanitation in public spaces	Inflation, revenue drops, unemployment in informal sector	National stimulus package, support for informal sector recovery with UNDP, creation of safer market spaces
Middle East & Arab Region			
Alexandria, Egypt	Strict traffic restrictions with phased reopening, limited public gatherings	Tourism revenue drop, unemployment rises in tourism	Tourism relief programs, health partnerships, and resilience strategies to boost economic activity in key sectors
Beirut, Lebanon	Intermittent traffic restrictions based on infection rate, closed non-essential businesses	Deep economic crisis worsened, high inflation	Temporary job creation, international aid, reform focus on essential services and infrastructure
Kuwait City, Kuwait	Curfews, restricted business hours, remote work protocols	GDP contraction, public sector job losses, migrant impact	Government relief packages, focus on supporting citizens and essential service continuity
Asia & Pacific			
Hoi An, Vietnam	Traffic restrictions, strict tourism and travel restrictions	Tourism revenue loss, SME closures	National stimulus, local tourism recovery efforts, SME support programs, digital marketing for tourism
Pune, India	Strict traffic restriction, mask mandates, travel restrictions	High unemployment in informal sector, limited mobility	Digital initiatives for essential services, partnerships for vaccine roll-out, aid to informal workers
Subang Jaya, Malaysia	Movement restrictions, business hour limits, public gathering bans	Retail sector affected, high impact on SMEs	Relief packages for SMEs, health campaigns, phased reopening, digitalization of public services
Europe			
Barcelona, Spain	Traffic restrictions with mobility restrictions, limited business operations	Major tourism revenue drop, increased unemployment	Revised budgets, public-private recovery partnerships, SME support, investment in public service digitalization
Bishkek, Kyrgyzstan	National traffic restriction, restricted public gatherings	Business closures, unemployment, disrupted supply chains	Local economic recovery plans, small business support, and partnerships with NGOs for health initiatives
Tirana, Albania	Curfews, limited public events, school closures	Small business strain, informal sector impacted	Micro-loan programs, SME support, focus on long-term economic resilience
Latin America & the Caribbean			
Guayaquil, Ecuador	Traffic restrictions, health checks at public facilities, restricted gatherings	High unemployment, health service strain, poverty increase	Partnership with private sector for health resources, economic relief for low-income communities, tourism support
Lima, Peru	Strict traffic restrictions, curfew, and movement restrictions	Loss in tourism revenue, rise in informal employment	Emergency support for food and health, SME aid programs, public health awareness campaigns
Teresina, Brazil	Traffic restrictions, restrictions on non-essential services	High impact on small businesses, reduced public revenue	Municipal budget reallocation, digital initiatives for service delivery, social support for informal workers

*Note:* * data refers to CRGP document [7]

*Abbreviations*: IGF, internally generated funds; GDP, gross domestic product; UNDP, United Nations Development Programme; NGO, non-governmental organization; SME, mall and mid-size enterprises.

African cities such as Harare, Zimbabwe implemented partnerships and aid programs whereas cities in Latin America like Guayaquil, Ecuador collaborated with the private sector to sustain health services and support low-income communities. Cities in the Middle East such as Alexandria, Egypt and Beirut, Lebanon, saw the government leverage international aid and local partnerships to support essential services. In the Asia and Pacific region, Hoi An, Vietnam and Pune, India promoted digital initiatives for essential services and launched economic aid programs to support small businesses and informal workers. Similarly, European cities such as Barcelona, Spain and Bishkek, Kyrgyzstan emphasized digital public service delivery, partnerships to support small and mid-size enterprises, and economic recovery planning [7].

During the later stage of the pandemic, the shift toward more targeted prevention and control measures was needed to transition back to normal. As outlined by the “10-point” and “20-point” strategies, China demonstrated a shift from blanket, population-wide interventions to more precise strategies that minimized disruption to daily life [8]. By focusing on high-risk areas and populations, health authorities were able to optimize resource allocation, target vaccination efforts, and adjust restrictions in real time, based on local conditions [9]. Such efforts enabled the government to efficiently address local needs while preserving economic stability.

The end of pandemic does not mean an end to health prevention. Public health systems must be strengthened and optimized according to their performance in the response phase, and risk factors must be continuously monitored to adequately prepare for a potential public health emergency.

## 4 Competency and innovation for infection prevention and control

The pandemic also revealed critical gaps in the training and competencies of professionals in infection prevention and control (IPC) worldwide [10]. A systematic review by Chen et al. [11] clarified how the COVID-19 pandemic pushed health institutions to reevaluate the core competencies needed in IPC roles— to not only be adept regarding clinical precautions, such as hand hygiene and infection source tracing, but also to take on leadership roles in program management, patient safety, and occupational health to integrate knowledge of microbiology with the practicalities of managing large-scale public health interventions.

The COVID-19 pandemic also accelerated the use of digital health and artificial intelligence (AI) solutions for infectious disease prevention and management to make up for shortages in health care workforces and competency deficiency. AI-driven solutions have facilitated effective pandemic forecasting, policy support, and drug discovery [12]. With respect to comorbidity management, online HIV treatment services have been adapted to pandemic-related constraints to maintain essential health services for people living with HIV, exemplifying the potential of telemedicine to complement traditional health care delivery models [13]. Although born of necessity during the pandemic, such innovations hold promise for enhancing health care access and equity in the long term, particularly in resource-limited settings where scientists and health professionals bear the brunt of policy failures and resource shortages [14].

## 5 Global health governance for a coordinated future

Experiences during the COVID-19 pandemic have highlighted the global nature of infectious disease threats and the importance of international collaboration in addressing them. Efforts has been made by the WHO to coordinate equitable vaccine distribution, as well as address global challenges in achieving vaccine access, equity, and justice in Africa and South America [15]. Experiences with COVID-19 in nearly 30 countries call for stronger global frameworks to support health system resilience, especially in under-resourced settings [16]. Strengthening these frameworks is essential to improving preparedness for future health crises.

## 6 Conclusion

The COVID-19 pandemic has revealed the crucial role of health policy, governance, and management in epidemic control. Although clinical and virological aspects of infectious disease outbreaks are essential, these must be complemented by strong public health leadership, responsive policy-making, and innovations in health care delivery. As global health systems evolve, lessons from COVID-19 provide a foundation for building more resilient, adaptable, and equitable health infrastructures.

## Funding

You Wu was supported by the Tsinghua University Start-up Fund (#53335000124).

## CRediT authorship contribution statement

**Zeyu Zhang:** Writing – original draft, Writing – review & editing. **You Wu:** Conceptualization, Writing – original draft, Writing – review & editing.
